# Peritoneal metastases of unknown primary with hepatoid features

**DOI:** 10.1515/pp-2022-0113

**Published:** 2022-08-19

**Authors:** Lakhdar Khellaf, Stéphanie Nougaret, Sébastien Carrère, Frédéric Bibeau

**Affiliations:** Departement of Pathology, Institut du Cancer de Montpellier (ICM) – Val d’Aurelle Montpellier Cedex, France; Departement of Radiology, Institut du Cancer de Montpellier (ICM) – Val d’Aurelle, Montpellier, France; Departement of Surgery, Institut du Cancer de Montpellier (ICM), Montpellier, France; Departement of Pathology, Centre Hospitalier Universitaire de Besançon, Besançon, France

**Keywords:** ectopic liver, hepatocellular carcinoma, hepatoid adenocarcinoma, sal-like protein 4

**Figure 1:**
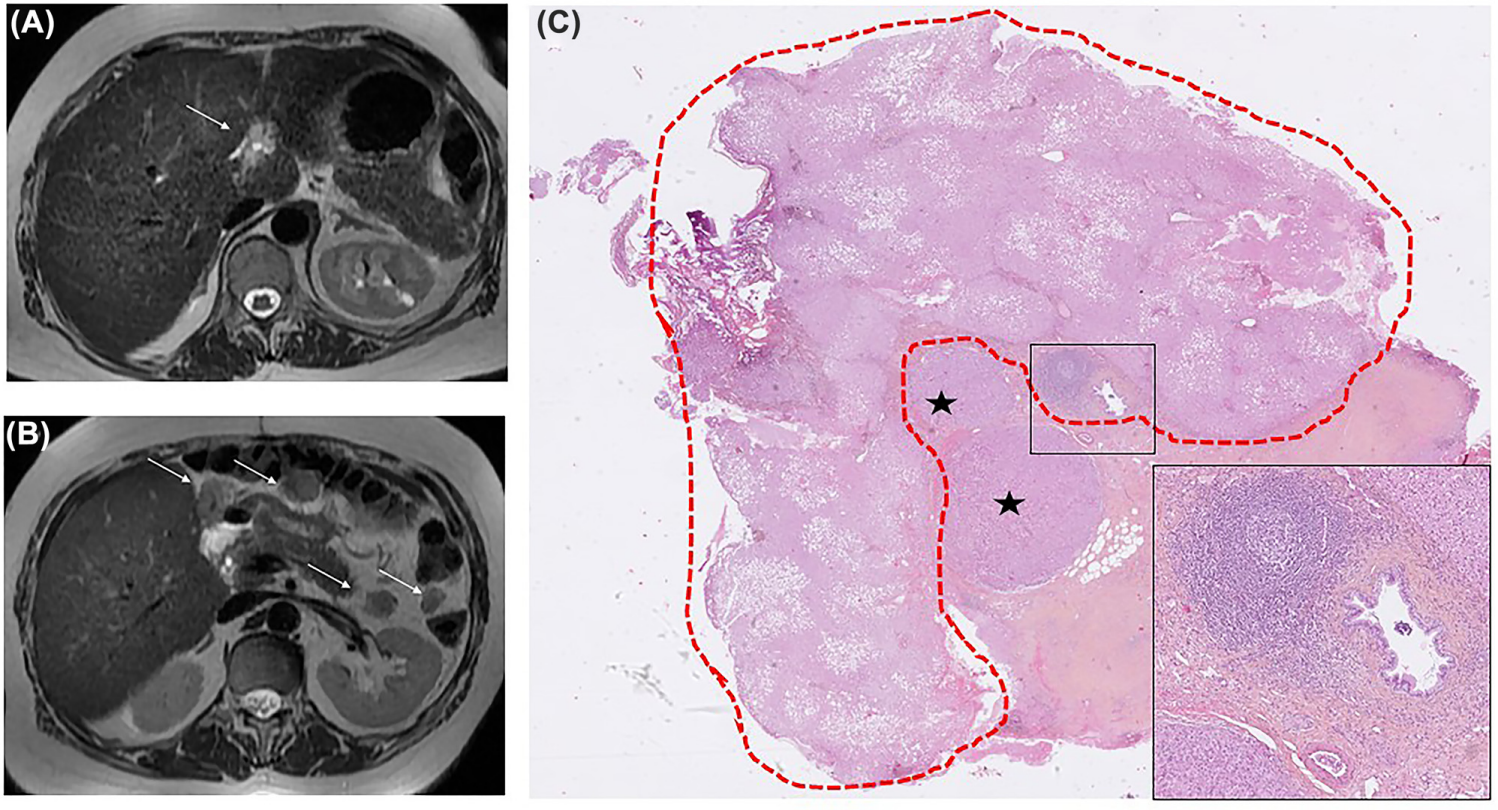
Magnetic resonance imaging (MRI) of the liver and peritoneum (axial T2 weighted images). (A) Initial MRI: ill-defined T2 hyperintense nodule within the falciform ligament of the liver (arrow). (B) MRI at 3 months: Appearance of several peritoneal metastases (arrows). Note the absence of any parenchymal liver tumour in both images. (C) Histopathological analysis: hepatocellular carcinoma (asterisks) in the falciform ligament of the liver, representing the starting point of the peritoneal disease. Ectopic liver is circled in red (greater axis: 15 mm), with detectable steatosis (HES, ×6). Note the independent vasculo-biliary stalk, highlighted in the inset (HES, ×50). HES: hematoxylin-eosin-saffron.

A 59-year-old woman presented with isolated peritoneal metastases in the context of elevated serum AFP levels (Figure 1A and B). No primary tumour was found, notably from the liver, the gastrointestinal or gynecological tracts. A laparoscopic assessment reported a peritoneal cancer index (PCI) reaching 22/39 and biopsies performed disclosed hepatocellular carcinoma (HCC). A chemotherapy followed by a cytoreductive surgery/hyperthermic intraoperative intra-peritoneal chemotherapy (HIPEC) was given, leading to a complete macroscopic clearance. Ultimately, liver metastases appeared, resulting in death.

All surgical specimens histologically showed features of HCC. An unsuspected ectopic liver (EL) was observed within the falciform ligament of the liver, in close vicinity of tumour (Figure 1C), strongly suggesting an EL cancerization [1, 2].

This unusual clinical presentation may first suggest a peritoneal extension from a liver HCC, which is usually encountered in massive-type tumours [3]. Rare hepatoid carcinomas can also be suspected in the context of a primary gastric tumour showing an adenocarcinomatous component with positive Sal-like protein 4 (SALL4) immunostaining [4].
